# Evolution and Antigenic Differentiation of Avian Influenza A(H7N9) Virus, China

**DOI:** 10.3201/eid3006.230530

**Published:** 2024-06

**Authors:** Yang Liu, Yuhua Chen, Zhiyi Yang, Yaozhong Lin, Siyuan Fu, Junhong Chen, Lingyu Xu, Tengfei Liu, Beibei Niu, Qiuhong Huang, Haixia Liu, Chaofeng Zheng, Ming Liao, Weixin Jia

**Affiliations:** South China Agricultural University, Guangzhou, China (Y. Liu, Z. Yang, Y. Lin, S. Fu, J. Chen, L. Xu, T. Liu, B. Niu, Q. Huang, M. Liao, W. Jia);; Guangzhou Animal Health Inspection Institute, Guangzhou (Y. Chen);; Guangdong Aib Polytechnic College, Guangzhou (H. Liu);; Animal Husbandry and Veterinary Station, Urumqi, China (C. Zheng)

**Keywords:** Viruses, zoonoses, avian influenza, H7N9, evolution, antigenic drift, influenza A virus, China

## Abstract

We characterized the evolution and molecular characteristics of avian influenza A(H7N9) viruses isolated in China during 2021–2023. We systematically analyzed the 10-year evolution of the hemagglutinin gene to determine the evolutionary branch. Our results showed recent antigenic drift, providing crucial clues for updating the H7N9 vaccine and disease prevention and control.

From early 2013 through October 2017, a total of 5 outbreaks of avian influenza A(H7N9) virus infection occurred, resulting in 616 human deaths (*1*). In particular, the fifth wave of the epidemic saw a substantial increase in human fatalities. By late 2017, a total of 1,568 laboratory-confirmed cases of H7N9 virus infection in humans had been reported according to International Health Regulations guidelines (https://www.who.int/emergencies/disease-outbreak-news/item/26-october-2017-ah7n9-china-en). The rapid emergence, prevalence, and pandemic potential of H7N9 virus were suddenly of great concern. Since 2017, low-pathogenicity avian influenza H7N9 virus transformed into the highly pathogenic avian influenza (HPAI) A(H7N9) virus (*2**–**5*). In response, China initiated a large-scale vaccination program in the poultry industry, effectively limiting the H7N9 epidemic. Although no human H7N9 infections have been reported since February 2019, the virus is still circulating in poultry, particularly in laying hens, and remains a potential threat to poultry industry and public health (*6**–**8*). Furthermore, since 2017, the H7N9 virus has undergone multiple instances of antigenic drift to evade immune pressure from vaccines (*9**–**11*). We investigated the genetic evolution and antigenic differentiation of the H7N9 virus in China to provide information to better control the epidemic, ensure the safety of the poultry industry, and protect public health.

## The Study

**Figure 1:**
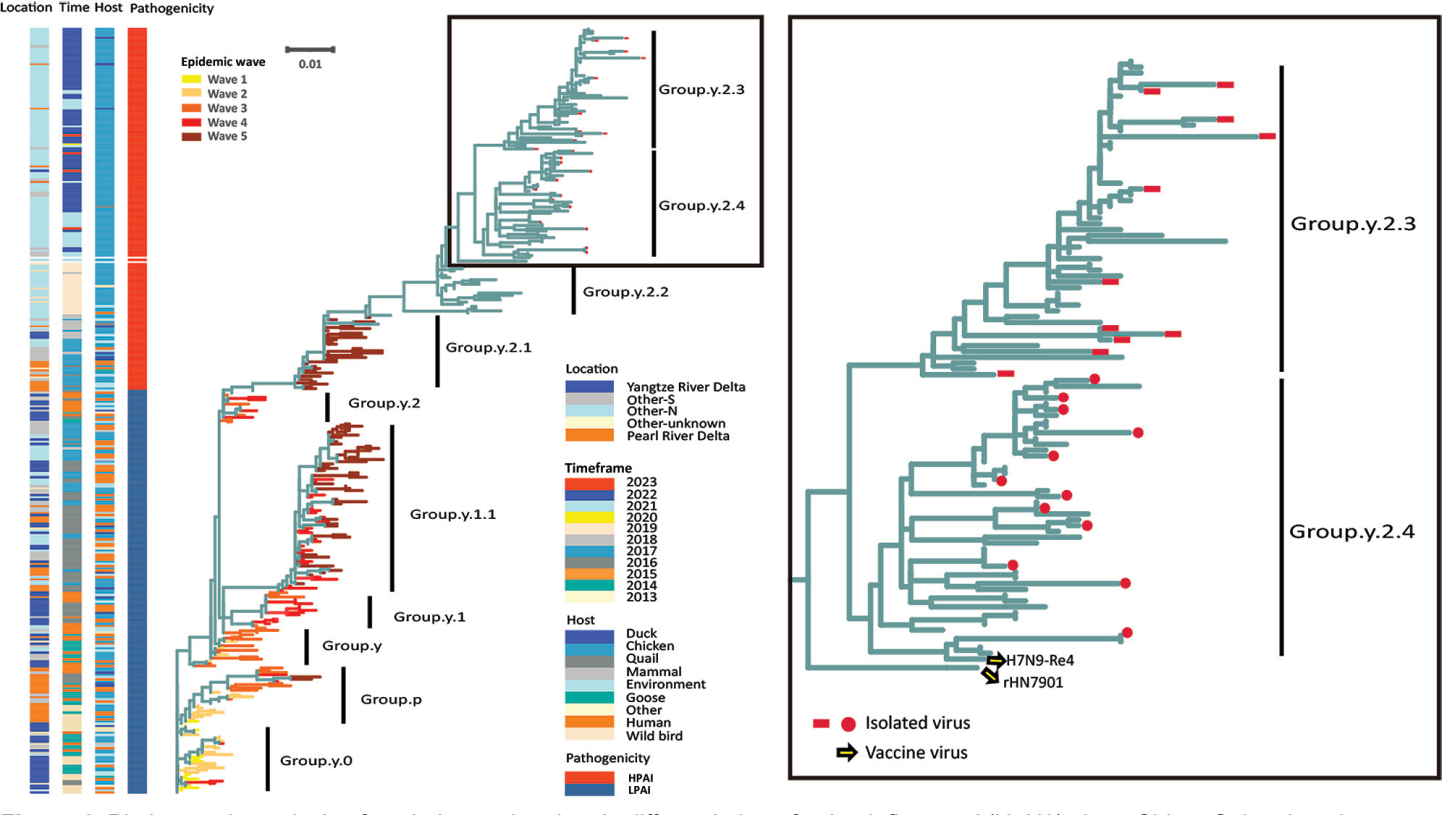
Phylogenetic analysis of evolution and antigenic differentiation of avian influenza A(H7N9) virus, China. Colors in columns at left show locations, timeframes, hosts, and pathogenicity of virus strains. The maximum-likelihood phylogenetic tree of the hemagglutinin gene depicts viruses corresponding to epidemic waves 1–5. Tree on right shows detail of Group.y.2.3 (red rectangles) and Group.y.2.4.4 (red circles) in comparison with vaccine strains. Scale bar indicates nucleotide substitutions per site. LPAI, low-pathogenicity avian influenza; HPAI, highly pathogenic avian influenza; Other-N, sites in the northern region; Other-S, sites in the southern region.

Through continuous monitoring of markets and breeding farms in several provinces, we successively isolated 23 H7N9 viruses. Using the sequences of those viruses and a reference sequence from the GISAID database (*12*), we conducted a phylogenetic analysis to study the evolution of H7N9 virus over the past decade (Figure 1).

 We rooted the maximum-likelihood phylogenetic tree with A/Anhui/1/2013 and identified the branches as Group.y.0. During 2013–2017, the 5 low-pathogenicity avian influenza H7N9 virus waves formed Group.y.0–Group.y.2 branch. The first wave was mainly prevalent in the Yangtze River Delta. In 2014, the second wave spread to the Pearl River Delta and gradually expanded to all parts of the country in the subsequent 3 waves. Around 2017, or even as early as mid-2016, researchers successfully isolated an HPAI variant of H7N9 virus (*6*). That variant was found to contain alkaline amino acids inserted into the cleavage site of the hemagglutinin protein (*6*,*13*). The discovery of that variant in live poultry markets in Guangdong Province indicated an increased pathogenicity to poultry and potentially posed a greater threat to human health. We found that those HPAI H7N9 virus variants clustered within the Group.y.2.1 branch and its subordinate branch. 

After implementation of a universal immunization program in 2017, the H7N9 virus outbreak was effectively controlled. However, some H7N9 viruses evolved to evade the vaccine. Those viruses continued to evolve and formed a new branch, Group.y.2.2, which is mainly found in northern China (Figure 1). Further investigation revealed that H7N9 virus is prevalent in the Bohai Rim region. Of note, we found no great differences in geographic, temporal, or host distribution between the 2 newly differentiated branches, Group.y.2.3 (A/Chicken/Hebei/1009/2020-like) and Group.y.2.4 (A/Chicken/Yunnan/1001/2021-like) (Figure 1). Those branches showed an average of 2.41% pairwise nucleotide distances between 2021 and 2023. That finding suggests that the evolutionary differences between those clades might not be influenced by geographic isolation, period, or host species, but rather by the adaptation of a new virus to natural selection. Positive selection pressure, which encourages mutations that contribute to the virus’ adaptation to the environment, can play a role in viral evolution. Our analysis confirmed that an increase in positive selection pressure in H7N9 virus occurred at some sites after 2017 (Appendix Table 1). 

**Figure 2:**
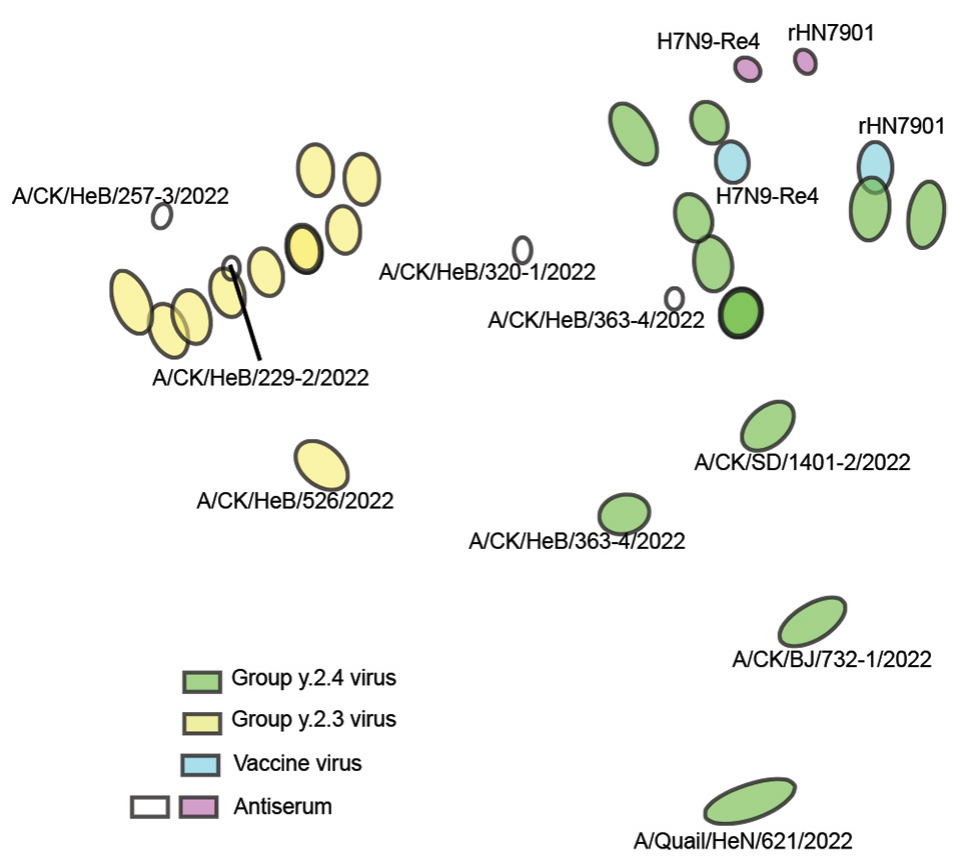
Antigenic map of avian influenza A(H7N9) virus, China, 2021–2023. The map was plotted using hemagglutinin inhibition assay results of 26 antigens (green, blue, and yellow dots), serum from 2 vaccine virus strains, H7N9-Re4 and rHN7901 (purple dots), and in-house designed serum of 4 circulating viruses (CK for chicken). The antigen map was constructed using the online website of the Antigenic Cartography Group, University of Cambridge (https://acmacs-web.antigenic-cartography.org). A/Chicken/HeB/257-3/2022 and A/Chicken/HeB/229-2/2022 belong to the Group.y.2.3 branch, whereas A/Chicken/HeB/320-1/2022 and A/Chicken/HeB/363-4/2022 belong to the Group.y.2.4 branch (indicated by white dots). The distance between the figures represents the antigen distance.

To examine whether mutation and evolution of H7N9 viruses are a result of antigenic drift and discontinuous variation, we used serologic methods to assess the antigenicity of the more evolved viruses from different clades (Appendix Table 2). Using the H7-Re4 and rHN7901 vaccine viruses for comparison, we found a weak cross-reaction titer between the vaccine viruses and the epidemic viruses in Group.y.2.3 (Table). The antigenic map demonstrated that the Group.y.2.3 viruses were distantly located from the vaccine serum (Figure 2), implying a consistent antigenic drift and greater antigenic divergence from Group.y.2.4 viruses. However, the distance between the vaccine virus and certain Group.y.2.4 viruses was relatively close (Figure 2), suggesting minimal differences. Furthermore, some Group.y.2.4 viruses, including A/Chicken/BJ/732-1/2022 and A/Quail/HeN/621/2022, both originating from northern China, also exhibited antigenic drift.

**Table:** Hemagglutination inhibition titers of 23 H7N9 epidemic viruses and vaccine viruses in a study of evolution and antigenic differentiation of avian influenza A(H7N9) virus, China*

Group	Antigen	Antiserum, log_2_					
		H7N9-Re4	rHN7901	229–2	257–3	320–1	363–4
Referent	H7N9-Re4	**10**	10	5	5	10	10
Referent	rHN7901	9	**10**	3	2	9	10
y.2.3	A/Chicken/HeB/229–2/2022	5	5	**10**	10	9	8
y.2.3	A/Chicken/HeB/257–3/2022	5	3	10	**10**	9	8
y.2.3	A/Quail/HeN/782–2/2022	4	4	10	9	8	6
y.2.3	A/Chicken/LN/976–3/2022	5	4	10	9	8	6
y.2.3	A/Duck/HeB/976–2/2022	5	4	10	9	7	5
y.2.3	A/Chicken/HeB/199–1/2022	7	5	10	10	9	8
y.2.3	A/Chicken/GX/J17/2022	7	7	10	10	8	9
y.2.3	A/Chicken/HeB/526/2022	6	5	8	8	7	7
y.2.3	A/Chicken/HeB/229–4/2022	6	6	10	10	9	8
y.2.3	A/Chicken/SX/B1323–1/2022	7	7	9	10	9	6
y.2.3	A/Chicken/SX/B22–2/2023	7	8	9	10	9	7
y.2.4	A/Chicken/HeB/363–4/2022	5	5	6	5	9	**10**
y.2.4	A/Chicken/HeB/320–1/2022	8	9	6	2	**10**	7
y.2.4	A/Quail/HeN/621/2022	5	3	3	4	6	6
y.2.4	A/Chicken/BJ/732–1/2022	5	5	5	3	6	9
y.2.4	A/Chicken/HuB/J15/2022	9	8	7	6	7	10
y.2.4	A/Chicken/BJ/470–6/2022	8	9	4	3	7	9
y.2.4	A/Chicken/SC/468–2/2022	8	8	6	5	8	10
y.2.4	A/Chicken/HeB/J94/2022	8	8	6	5	8	10
y.2.4	A/Chicken/YN/415–2/2022	9	9	8	6	8	9
y.2.4	A/Chicken/SD/1401–2/2021	7	7	6	4	8	9
y.2.4	A/Chicken/JSu/B14–3/2023	9	9	6	6	9	10
y.2.4	A/Chicken/HeB/B14–1/2023	10	10	6	6	9	9

*Bold text indicates the cross titers of sera with corresponding antigens. BJ, Beijing; GX, Guangxi; HeB, Hebei; HeN, Henan; HuB, Hubei; JS, Jiangsu; LN, Liaoning; SC, Sichuan; SD, Shandong; SX, Shanxi; YN, Yunnan.

Changes in antigenicity often are caused by accumulation of amino acid mutations in antigenic sites. Therefore, we compared virus sequences and observed that the cleavage sites were KRKRTAR↓GLF or KRKRIAR↓GLF, both of which exhibited the characteristics of HPAI viruses. However, we noted no substantial differences between Group.y.2.3 and Group.y.2.4 at positions 86, 129, 134, 141, 145, 148, 151, 159, 208, 284, and 319 of H7 (Appendix Table 3). Those findings demonstrated the high genetic diversity of the H7N9 virus. Except for position 208 in H7, all sites were antigenic sites, and positions 141, 145, and 148 were both antigenic sites and receptor-binding sites. For the Group.y.2.4 branch, we compared the hemagglutinin 1 peptide of the vaccine viruses against antigenically distant viruses A/Chicken/SD/1301-2/2022, A/Chicken/HeB/B363-4/2022, A/Chicken/BJ/B732-1/2022, and A/Quail/HeN/621/2022. We observed different amino acids that could affect H7N9 virus antigenicity (Appendix Table 4). Among the analyzed viruses, A/Quail/HeN/621/2022 exhibited the highest number of mutations compared with the vaccine viruses, followed by A/Chicken/BJ/B732-1/2022 and A/Chicken/HeB/B363-4/2022; A/Chicken/SD/1401-2/2022 displayed the fewest mutations. Moreover, the previously reported Q226L and G228S sites of H3 viruses (Appendix Table 5), which have the potential to enhance mammalian adaptation, remained unchanged in all H7N9 viruses. Those sites still showed a preference for avian receptors, except A/Quail/HeN/621/2022, which mutated to P at position 160. All V125T H3 sites were replaced, indicating that the receptor-binding capacity and immune escape of the virus might be affected, making the virus more compatible with avian receptors (*14*,*15*).

## Conclusions

This study explored the evolution and antigenic differentiation characteristics of H7N9 virus over the past decade through continuous monitoring and selection of representative sequences from all publicly available H7N9 virus sequences. However, our research still had certain limitations, and further investigation is needed to understand the relationship between the evolution of viruses under positive selection pressure and the underlying cause of antigenic variation. 

In summary, influenza A viruses are highly prone to mutation and evolution, making the H7N9 virus epidemic more complex and challenging to control. This study offers vital insights into the genetic evolutionary branches and recent antigenic drift, providing crucial clues for updating the H7N9 vaccine seed virus and for disease prevention and control.

Appendix Additional information on evolution and antigenic differentiation of avian influenza A(H7N9) virus, China.
