# Supplementary material for: Evolution and Antigenic Differentiation of Avian Influenza A(H7N9) Virus, China
Source: Emerg Infect Dis. 2024 Jun;30(6):1218–22. doi: 10.3201/eid3006.230530 (PMC11138980; doi:10.3201/eid3006.230530)
Supplement: Appendix — Additional information on evolution and antigenic differentiation of avian influenza A(H7N9) virus, China. [file 23-0530-Techapp-s1.pdf]

Article DOI: https://doi.org/10.3201/eid3006.230530

EID cannot ensure accessibility for supplementary materials supplied by authors. Readers who have difficulty accessing supplementary content should contact the authors for assistance.

Evolution and Antigenic Differentiation of Avian Influenza A(H7N9) Virus, China

Appendix

Materials and Methods

Sample Collection and Virus Isolation

Some of the samples consisted of respiratory, throat, and cloacal swabs collected regularly from live poultry markets in Guangdong, and others collected during surveillance of poultry farms in multiple provinces in China between October 2021 and February 2023. Samples were stored in a 2 mL PBS sampling solution containing 5,000 units/L penicillin and 5,000 units/L streptomycin mixed with 10% glycerin, transported to the laboratory using cold chain logistics and stored in a –80°C freezer. The samples are then inoculated into the allantoic cavity of 9- to 11-day-old embryonated chicken eggs. The eggs are incubated at 37°C for 48–72 hours. The allantoic fluid of any eggs containing dead or dying embryos during the incubation and all eggs at the end of the incubation period are tested for the presence of hemagglutinating activity. Two types of methods were used to identify the virus: hemagglutination inhibition tests was employed in diagnostic serology; the genome of specific subtypes is identified using RT-PCR, sequencing, and phylogenetic analysis. Then the mixed virus was purified by plaque method. (Detailed methods for virus isolation and identification followed the *Manual of Diagnostic Tests and Vaccines for Terrestrial Animals*, twelfth edition, 2023 published by the World Organization for Animal Health.) We have tested a total of 2,785 samples, and no positive samples of the

H7N9 virus were found in the live poultry market in Guangdong; all positive samples came from non-diseased poultry on farms.

Sequencing of Samples

RNA was extracted from HA-positive samples using reagents and methods according to the manufacturer's instructions (Fastagen Biotech, Shanghai, China), including RNA inhibition degradation enzyme (RRI enzyme), M-MLV reverse transcription buffer, dNTPs, Premix Ex Taq, DL1000 Marker, and DL2000 Marker (TaKaRa Bio). PCR was performed using the universal primers described by Hoffmann et al (*1*). PCR products were purified with a QIAamp Gel extraction kit (Qiagen) and sequenced with an ABI 3730XL DNA Analyzer (Applied Biosystems).

Genetic Evolution Analysis and Cladistic Division

In this study, 2,544 complete HA sequences collected in Asia from January 2013 to March 2021 were downloaded from the GISAID database (http://www.gisaid.org). The MAFFT (v7.273) program was used for comparison (*2*) and incomplete ORF sequences were removed. Data with similar attributes were screened, and representative viruses were selected as references combined with time, host, and region information. Then 343 viruses were analyzed for phylogenetic analysis combined with the sequences previously published in our laboratory, the latest virus isolated from 2022 to 2023 and vaccine virus sequence. IQ-TREE (*3*) was built using PhyloSuite v1.2.2 . The maximum likelihood phylogeny was inferred under a 5,000 ultrafast bootstrap combined with the GTR+I+G4+F model, and visualized and polished using the online Interactive Tree of Life (iTOL) software. We referred to the WHO/FAO/OIE basis for the division of H5AIV clades (*4*) and used MEGA to calculate the nucleotide genetic distance difference fractions for some clades.

In our study, the following steps were followed to systematically analyze and name the evolution of viruses: (i) Average percentage pairwise nucleotide distances between and within

clades of >1.5% and <1.5%; (ii) Combine the geographic features of the H7 viruses from 2013 to 2017 — the virus originated in the Yangtze River Delta, spread to the Pearl River Delta, formed two major foci, and finally spreading the Northern provinces; (iii) Combine the temporal characteristics of H7 viruses from 2013 to 2017 — the regularity of an annual epidemic; (iv) If the average percentage pairwise nucleotide distances between clades in (i) is less than 1.5%, but the characteristics in (ii) and (iii) of the virus are significantly different, the clade is then assigned to a different clade.

Based on those steps, the naming system for H7N9 was proposed, which is illustrated in Figure 1. Branch is defined as follows: The LP H7N9 AI first emerged broke out in the Yangtze River Delta (Group.y.0) in 2013, and then rapidly spread to the Pearl River Delta (Group.p). In 2014, Group.y.0 evolved into Group.y and Group.y.1. The Group.y.1 clade contained the virus from the Yangtze River Delta. It soon spread to other southern and northern parts of China and gathered into the ML tree Group.y.1.1 branch. Simultaneously, Group.y evolved a clade Group.y.2, which was different from that of the Group.y.1 After 2017, through a series of mutations, modifications, reassertions, and rearrangements, cunning viruses continued to evade immunity and evolved into the Group.y.2.2. Due to a lack of data, we do not have much information on the evolution of viruses from 2019 to 2020. After 2021, our detection data showed that Group.y.2.2 further differentiated into Group.y.2.3 and Group.y.2.4 subbranches.

Selection and Adaptive Evolution of Genetic Variation

Natural selection is a basic process that affects population evolution. We used the online Datamonkey Web site to estimate the ratio of the nonsynonymous replacement rate to the synonymous replacement rate (dN/dS) of nucleotide sequences of the A(H7N9) virus from 2013 to 2017 and 2018 to 2023. (dN/dS(ω)) can either be $\omega \approx 1.0$, $\omega > 1.0$, or $\omega < 1.0$, indicating neutral, positive, or negative selection, respectively. MEME, FUBAR, and SLAC were used to detect the selection pressure of the HP H7N9 virus (sequences containing stop codons and not divisible by 3 could not be read). MEME model assumes that the selection pressure of each site is universal

or sporadic throughout the phylogenetic tree (*5–7*), and MEGA was used to visualize the sequence and count the differences in amino acid sites.

Preparation of Antisera, Hemagglutination Inhibition Assay, and Antigen Mapping

After the virus was proliferated in chicken embryos, 30%–40% formaldehyde was used for inactivation at 37°C for 48 h. After the inactivation and the sterility tests, the same amount of adjuvant was added the antigen to prepare the vaccine. Two 4-week-old SPF chickens (male and female) were used for each antigen test. Each chicken was intramuscularly injected with 0.5 mL homemade antigen (0.5 mL dispersed into the pectoralis major and leg muscles for injection), followed by a second 0.5 mL intramuscular injection after 4–5 days, and a third 0.5 mL intramuscular injection after 4–5 days. The cardiac blood was collected to harvest the serum after 3–4 weeks Antibody titers were determined after 12 days. The hemagglutination inhibition test was performed in accordance with the SOP prescribed by the World Organisation for Animal Health (WOAH). HI endpoint titers were determined as the highest serum dilution that completely inhibited HA activity; thus, the unit was Log2. The results of HI crossing were visualized on an antigen map using the ACMACS Web site (*8*). All animal experiments were reviewed and approved by the Laboratory Animal Ethics Committee, South China Agricultural University, and were carried out under the approved guidelines (approval no. 2023B018).

References

1. Hoffmann E, Stech J, Guan Y, Webster RG, Perez DR. Universal primer set for the full-length amplification of all influenza A viruses. Arch Virol. 2001;146:2275–89. PubMed https://doi.org/10.1007/s007050170002

2. Katoh K, Misawa K, Kuma K, Miyata T. MAFFT: a novel method for rapid multiple sequence alignment based on fast Fourier transform. Nucleic Acids Res. 2002;30:3059–66. PubMed https://doi.org/10.1093/nar/gkf436

3. Zhang D, Gao F, Jakovlić I, Zou H, Zhang J, Li WX, et al. PhyloSuite: an integrated and scalable desktop platform for streamlined molecular sequence data management and evolutionary phylogenetics studies. Mol Ecol Resour. 2020;20:348–55. PubMed https://doi.org/10.1111/1755-0998.13096

4. World Health Organization/World Organisation for Animal Health/Food and Agriculture Organization H5N1 Evolution Working Group. Toward a unified nomenclature system for highly pathogenic avian influenza virus (H5N1). Emerg Infect Dis. 2008;14:e1. PubMed https://doi.org/10.3201/eid1407.071681

5. Weaver S, Shank SD, Spielman SJ, Li M, Muse SV, Kosakovsky Pond SL. Datamonkey 2.0: a modern web application for characterizing selective and other evolutionary processes. Mol Biol Evol. 2018;35:773–7. PubMed https://doi.org/10.1093/molbev/msx335

6. Pond SL, Frost SDW. Datamonkey: rapid detection of selective pressure on individual sites of codon alignments. Bioinformatics. 2010;10:2531–3. PubMed https://doi.org/10.1093/bioinformatics/bti320

7. Delport W, Poon AFY, Frost SDW, Kosakovsky Pond SL. Datamonkey 2010: a suite of phylogenetic analysis tools for evolutionary biology. Bioinformatics. 2010;26:2455–7. PubMed https://doi.org/10.1093/bioinformatics/btq429

8. Smith DJ, Lapedes AS, de Jong JC, Bestebroer TM, Rimmelzwaan GF, Osterhaus AD, et al. Mapping the antigenic and genetic evolution of influenza virus. Science. 2004;305:371–6. PubMed https://doi.org/10.1126/science.1097211

Appendix Table 1. Positive selection pressure site of HA gene in H7N9 virus

	Mode		
Timeframe	MEME p<0.1	FUBAR Prob>0.9	SLAC p<0.1
2013–2017	65/29/5/276	65	65
2018–2023	12/13/65/134/143/145/284/563/564	12/13/65/134/136/148/151/152/182/335/563	65/134

*FUBAR, Fast, Unconstrained Bayesian AppRoximation; MEME, Mixed Effects Model of Evolution; SLAC, Single-Likelihood Ancestor Counting.

Appendix Table 2. Information on the viruses used in the cross-hemagglutinin inhibition assays

Isolate name	Host	Collection date	Location
A/Chicken/SD/1401–2/2021	Chicken	2021 Nov 16	Shandong
A/Chicken/HeB/199–1/2022	Chicken	2022 Mar 3	Hebei
A/Chicken/HeB/229–2/2022	Chicken	2022 Mar 10	Hebei
A/Chicken/HeB/229–4/2022	Chicken	2022 Mar 10	Hebei
A/Chicken/HeB/257–3/2022	Chicken	2022 Mar 23	Hebei
A/Chicken/HeB/320–1/2022	Chicken	2022 Apr 14	Hebei
A/Chicken/HeB/363–4/2022	Chicken	2022 Apr 28	Hebei
A/Chicken/YN/415–2/2022	Chicken	2022 May 6	Yunnan
A/Chicken/SC/468–2/2022	Chicken	2022/5/25	Sichuan
A/Chicken/BJ/470–6/2022	Chicken	2022/5/19	Beijing
A/Chicken/HeB/526/2022	Chicken	2022 Jun 2	Hebei
A/Quail/HeN/621/2022	Quail	2022 Jun 23	Henan
A/Chicken/BJ/732–1/2022	Chicken	2022 Jul 27	Beijing
A/Quail/HeN/782–2/2022	Quail	2022 Aug 4	Henan
A/Duck/HeB/976–2/2022	Duck	2022 Oct 27	Hebei
A/Chicken/LN/976–3/2022	Chicken	2022 Oct 27	Liaoning
A/Chicken/HuB/J15/2022	Chicken	2022 Jan 25	Hubei
A/Chicken/GX/J17/2022	Chicken	2022 Jan 25	Guangxi
A/Chicken/HeB/J94/2022	Chicken	2022 Apr 7	Hebei
A/Chicken/SX/B1323–1/2022	Chicken	2022 Dec 15	Shanxi
A/Chicken/SX/B22–2/2023	Chicken	2023 Feb 1	Shanxi
A/Chicken/JSu/B14–3/2023	Chicken	2023 Jan 17	Jiangsu
A/Chicken/HeB/B14–1/2023	Chicken	2023 Jan 17	Hebei

Appendix Table 3. Amino acid differential sites of different branching viruses

Group	Antigen and receptor binding sites	Amino acid differences (H7 number)												
		A	D	D	A	RBS/A	RBS/A	RBS/A	A	A	A	–	C	C
		86	111	129	134	141	145	148	150	151	159	208	284	319
Referent	rHN7901	Q	V	E	K	N	S	K	S	G	K	N	A	R
Referent	H7N9-Re4	Q	V	E	K	N	S	M	S	R	K	D	A	R
y.2.4	A/Chicken/JSu/B14–3/2023	Q	V	K	R	N	S	M	S	R	R	D	A	K
y.2.4	A/Chicken/HeB/B14–1/2023	Q	V	K	K	N	S	M	S	R	K	N	A	K
y.2.4	A/Quail/HeN/621/2022	Q	V	K	N	N	S	M	S	R	K	N	P	K
y.2.4	A/Chicken/SC/468–2/2022	Q	V	K	R	N	S	M	S	R	R	D	A	K
y.2.4	A/Chicken/SD/1401–2/2022	Q	V	K	Q	N	S	M	S	R	K	N	A	K
y.2.4	A/Chicken/HuB/J15/2022	Q	M	K	K	N	S	M	S	R	R	N	A	K
y.2.4	A/Chicken/HeB/320–1/2022	Q	V	K	N	N	S	M	S	R	K	N	A	K
y.2.4	A/Chicken/HeB/J94/2022	Q	V	K	R	N	S	M	S	R	R	D	A	K
y.2.4	A/Chicken/YN/415–2/2022	Q	V	K	E	N	S	M	S	R	K	D	A	R
y.2.4	A/Chicken/HeB/363–4/2022	Q	V	K	K	N	S	M	S	R	R	N	A	K
y.2.4	A/Chicken/BJ/470–6/2022	Q	V	K	R	N	S	M	S	R	R	D	A	K
y.2.4	A/Chicken/BJ/732–1/2022	Q	V	K	R	N	S	M	S	G	R	D	A	K
y.2.3	A/Chicken/SX/B22–2/2023	Q	V	E	E	N	R	R	T	E	K	N	A	R
y.2.3	A/Quail/HeN/782–2/2022	Q	V	E	K	N	R	R	T	E	K	N	A	R
y.2.3	A/Chicken/SX/B1323–1/2022	K	M	E	E	D	R	R	T	E	K	N	P	R
y.2.3	A/Chicken/HeB/526/2022	K	M	E	E	N	R	R	T	E	K	N	P	R
y.2.3	A/Chicken/HeB/229–4/2022	Q	M	E	E	D	R	R	T	E	K	N	P	R
y.2.3	A/Chicken/HeB/229–2/2022	K	M	E	E	D	R	R	T	E	K	N	P	R
y.2.3	A/Chicken/HeB/257–3/2022	K	M	E	E	D	R	R	T	E	K	N	P	R
y.2.3	A/Chicken/GX/J17/2022	Q	V	E	I	N	R	R	T	E	K	N	A	R
y.2.3	A/Chicken/LN/976–3/2022	K	M	E	E	D	R	R	T	E	K	N	P	R
y.2.3	A/Duck/HeB/976–2/2022	K	M	E	E	D	R	R	T	E	K	N	P	R
y.2.3	A/Chicken/HeB/199–1/2022	K	M	E	E	D	R	R	T	E	K	N	A	R

Appendix Table 4. Amino acid characteristics of Group.y.2.4 antigenically distant viruses and vaccines*

Cleavage sites	Re/4 KRKRTAR↓GLF	rHN7903/HA KGR↓GLF	A/Chicken/SD/1401–2/2022 KRKRTAR↓GLF	A/Chicken/HeB/B363–4/2022 KKKRTAR↓GLF	A/Chicken/BJ/B7 32–1/2022 KKKRTAR↓GLF	A/Quail/HeN/621/2022 KRKRIAR↓GLF
71	R	K	Q	Q	Q	Q
89	K	K	–	–	–	T
100	E	E	–	–	–	G
102	S	S	–	N	D	–
110	F	V	F	F	F	F
119	I	V	I	I	I	I
129	E	E	K	K	K	K
134	K	K	Q	–	R	N
136	D	N	D	D	N	D
148	M	K	M	M	M	M
151	R	G	R	R	G	R
152	S	P	S	S	S	S
159	K	K	–	R	R	–
164	N	D	N	N	N	N
165	T	T	–	–	–	K
169	T	T	–	–	–	P
182	E	E	–	–	–	G
208	D	N	N	N	D	N
214	E	E	–	–	–	G
231	K	Q	Q	Q	Q	Q
247	N	N	S	–	–	–
255	S	S	–	N	N	–
284	A	A	–	–	–	P
306	A	A	–	–	–	P
319	R	R	K	K	K	K

*– indicates that the amino acid of the virus is identical to that of the 2 vaccine viruses.

Appendix Table 5. Amino acid characteristics of key mutations

		Amino acid characteristics (H3 number)			
Virus group	Virus identification	V125T	T160A	Q226L	G228S
Referent	rHN7901	T	T	Q	G
Referent	H7N9-Re4	T	T	Q	G
y.2.4	A/Chicken/JSu/B14–3/2023	T	T	Q	G
y.2.4	A/Chicken/HeB/B14–1/2023	T	T	Q	G
y.2.4	A/Quail/HeN/621/2022	T	P	Q	G
y.2.4	A/Chicken/SC/468–2/2022	T	T	Q	G
y.2.4	A/Chicken/SD/1401–2/2022	T	T	Q	G
y.2.4	A/Chicken/HuB/J15/2022	T	T	Q	G
y.2.4	A/Chicken/HeB/320–1/2022	T	T	Q	G
y.2.4	A/Chicken/HeB/J94/2022	T	T	Q	G
y.2.4	A/Chicken/YN/415–2/2022	T	T	Q	G
y.2.4	A/Chicken/HeB/363–4/2022	T	T	Q	G
y.2.4	A/Chicken/BJ/470–6/2022	T	T	Q	G
y.2.4	A/Chicken/BJ/732–1/2022	T	T	Q	G
y.2.3	A/Chicken/SX/B22–2/2023	T	T	Q	G
y.2.3	A/Quail/HeN/782–2/2022	T	T	Q	G
y.2.3	A/Chicken/SX/B1323–1/2022	T	T	Q	G
y.2.3	A/Chicken/HeB/526/2022	T	T	Q	G
y.2.3	A/Chicken/HeB/229–4/2022	T	T	Q	G
y.2.3	A/Chicken/HeB/229–2/2022	T	T	Q	G
y.2.3	A/Chicken/HeB/257–3/2022	T	T	Q	G
y.2.3	A/Chicken/GX/J17/2022	T	T	Q	G
y.2.3	A/Chicken/LN/976–3/2022	T	T	Q	G
y.2.3	A/Duck/HeB/976–2/2022	T	T	Q	G
y.2.3	A/Chicken/HeB/199–1/2022	T	T	Q	G

Appendix Table 6. The accession number of HA gene of H7N9 viruses isolated from China during the period 2021–2023

Name	Segment	Accession
A/Chicken/HeB/363–4/2022	HA	EPI_ISL_18042586
A/Chicken/YN/415–2/2022	HA	EPI_ISL_18042620
A/Chicken/SC/468–2/2022	HA	EPI_ISL_18042621
A/Chicken/BJ/470–6/2022	HA	EPI_ISL_18042622
A/Chicken/HeB/526/2022	HA	EPI_ISL_18042623
A/Quail/HeN/621/2022	HA	EPI_ISL_18042624
A/Chicken/BJ/732–1/2022	HA	EPI_ISL_18042625
A/Quail/HeN/782–2/2022	HA	EPI_ISL_18042626
A/Duck/HeB/976–2/2022	HA	EPI_ISL_18042627
A/Chicken/LN/976–3/2022	HA	EPI_ISL_18042628
A/Chicken/HuB/J15/2022	HA	EPI_ISL_18042629
A/Chicken/GX/J17/2022	HA	EPI_ISL_18042630
A/Chicken/HeB/J94/2022	HA	EPI_ISL_18042631
A/Chicken/SX/B1323–1/2022	HA	EPI_ISL_18042638
A/Chicken/SX/B22–2/2023	HA	EPI_ISL_18042650
A/Chicken/JSu/B14–3/23	HA	EPI_ISL_18042665
A/Chicken/HeB/B14–1/23	HA	EPI_ISL_18042687
A/Chicken/HeB/320–1/2022	HA	EPI_ISL_18042581
A/Chicken/HeB/199–1/2022	HA	EPI_ISL_17445505
A/Chicken/HeB/229–2/2022	HA	EPI_ISL_18042577
A/Chicken/SD/1401–2/2022	HA	EPI_ISL_18042578
A/Chicken/HeB/229–4/2022	HA	EPI_ISL_18042579
A/Chicken/HeB/257–3/2022	HA	EPI_ISL_18042580
